# Evaluation of conjunctival inflammatory status by confocal scanning laser microscopy and conjunctival brush cytology in patients with atopic keratoconjunctivitis (AKC)

**Published:** 2009-08-15

**Authors:** Tais Hitomi Wakamatsu, Naoko Okada, Takashi Kojima, Yukihiro Matsumoto, Osama M.A. Ibrahim, Murat Dogru, Enrique Sato Adan, Kazumi Fukagawa, Chikako Katakami, Kazuo Tsubota, Jun Shimazaki, Hiroshi Fujishima

**Affiliations:** 1 Department of Ophthalmology, Keio University School of Medicine, Tokyo, Japan; 2 Johnson & Johnson Ocular Surface and Visual Optics Department, Keio University School of Medicine, Tokyo, Japan; 3 Department of Ophthalmology, Tokyo Dental College, Chiba, Japan; 4 Department of Ophthalmology, Kobe Kaisei Hospital, Kobe, Japan; 5 Department of Ophthalmology, International University of Welfare Mita Hospital, Tokyo, Japan

## Abstract

**Purpose:**

To elucidate the status of the conjunctival inflammation in atopic keratoconjunctivitis (AKC) using laser scanning confocal microscopy and compare the relevant findings with conjunctival brush cytology in a prospective controlled study.

**Methods:**

Twenty eyes from 20 AKC patients as well as 16 eyes from 16 age and sex matched normal subjects were studied. The subjects underwent tear film break-up time (BUT), fluorescein and Rose Bengal staining of the ocular surface, conjunctival confocal microscopy, Schirmer test, and brush cytology. Brush cytology specimens and in vivo confocal microscopy scans underwent evaluation for inflammatory cell densities.

**Results:**

Brush cytology specimens and in vivo confocal microscopy scans from AKC patients revealed significantly higher numbers of inflammatory cells (p<0.05). Conjunctival inflammatory cell density showed a negative correlation with tear stability and a positive correlation with vital staining scores and conjunctival injection grades. The extent of conjunctival inflammation assessed by in vivo confocal microscopy showed a strong positive linear correlation with the inflammation status evaluated by brush cytology. The corneal inflammatory cell density assessed by in vivo confocal microscopy showed a significant negative correlation with tear stability and a positive linear correlation with corneal fluorescein staining.

**Conclusions:**

Confocal scanning laser microscopy is an efficient, noninvasive, and a promising tool for the quantitative assessment of conjunctival inflammation, a parameter of this new technology which correlated well with subjective and objective ocular surface clinical findings.

## Introduction

Atopic diseases constitute an important public health problem in many societies. A community based sample of 8,206 adults aged 27–56 years, in 25 European centers showed higher prevalences in Scandinavia and UK with intermediate figures in western Europe [1]. Recent studies report the lifetime prevalence rates for the atopic diseases in children and adolescents as between 24%–45% [2].

Atopic keratoconjunctivitis (AKC) is a bilateral chronic hypersensitivity disease of the ocular surface associated with systemic atopic dermatitis (AD). The ocular inflammatory process and release of allergic mediators onto the ocular surface and tear film are thought to be responsible for a wide range of clinical corneal and conjunctival manifestations including superficial punctate keratitis, macroerosions, corneal ulceration, plaque formation, dry eyes, corneal neovascularization, lipid infiltration, conjunctival papillary hypertrophy, cicatrization, and symblepharon [3,4].

It is important to be able to assess and quantify the conjunctival inflammatory status in AKC for diagnostic reasons and to observe effects of treatment. Current methods which are available to diagnose and evaluate the ocular surface inflammation status include clinical assessment and grading of conjunctival injection by slit lamp microscopy, determination of tear inflammatory cytokines by ELISA, flow cytometry, conjunctival brush cytology, and biopsy [5-13]. Brush cytology is a useful but an invasive diagnostic method for quantitative determination of conjunctival inflammatory cell numbers in patients with ocular allergies.

Confocal microscopy is a new emerging non-invasive technology which is useful as a supplementary diagnostic tool for the in vivo assessment of the histopathology of many ocular surface diseases and anterior-segment disorders including the in-vivo examination of the bulbar and palpebral conjunctiva [14-20]. Confocal scanning laser microscopy was reported to be an efficient tool for the quantitative assessment of conjunctival inflammation, and evaluation of pathological alterations in the papillary lesions and their relation with the ocular surface disease in patients with AKC [21].

In this study, we evaluated the conjunctival inflammation and ocular surface status in AKC by confocal microscopy and brush cytology and compared the results with those of healthy control subjects. We also investigated the correlation of inflammatory cell numbers between the two techniques and studied the relationship of conjunctival inflammatory status to the clinical ocular surface findings and tear functions assessed both by confocal microscopy and brush cytology.

## Methods

### Subjects

Twenty right eyes of 20 AKC patients with atopic dermatitis (AD; 12 males, 8 females, age range: 9–43 years; average age: 20.3 years, mean ocular disease duration: 18 years) as well as 16 right eyes of 16 age and sex matched healthy normal subjects (9 males, 7 females, age range: 14–40 years; average age: 24.4 years) were studied. As the ethic board committee did not allow a wash-out period in subjects with an active disease process to study the naïve ocular surface status, all patients were being treated with topical 0.025% ketotifen fumarate q.i.d. and topical 0.01% betamethasone q.i.d. for 8 weeks at the time of the conduct of this study.

Patients who strictly adhered to their treatment regimens including the frequency of instillations but were refractory to the above protocol were recruited and underwent examination of tear functions and ocular surface health parameters before institution of additional treatment. Confocal scanning laser microscopy examinations were performed by the same researcher . All AKC patients had active AD. AD patients with symptoms of allergic conjunctivitis without seasonal aggravation, presenting with conjunctival papillae and keratopathy, were diagnosed as having atopic keratoconjunctivitis (AKC). Venous peripheral blood was collected and ELISA test to detect the specific IgE antibodies to 26 allergens was performed using the MAST 26 Allergen Kit in all subjects. (SRL, Tokyo, Japan). Those patients who had any history of Stevens-Johnson syndrome, chemical, thermal, or radiation injury, keratoconus, ocular or systemic disease other than AKC, a history of ocular surgery, or contact lens or drug use that would alter the ocular surface were excluded. None of the patients were being treated with systemic steroids, prostaglandin inhibitors, or systemic immunosuppressants at the time of inclusion into the study.

A conventional slit-lamp microscopic examination was performed. Severity of conjunctival injection was graded on a four point scale as described previously. Briefly, absence of injection was graded as zero and mild injection was graded as one point. Moderate injection with edema of the palpebral conjunctiva and hazy view of the deep tarsal vessels was given two points. Severe injection obscuring visualization of the deep tarsal vessels was graded as three points [22]. The subjects then underwent tear function and ocular surface examinations including tear film break-up time (BUT) measurements, fluorescein and Rose Bengal staining of the ocular surface, Schirmer test I, confocal scanning laser microscopy, and finally brush cytology of the upper palpebral conjunctiva. This research followed the tenets of the Declaration of Helsinki. Informed consent from all subjects were obtained.

### Tear function tests and ocular surface vital staining

The standard tear film break-up time measurement was performed. The ocular surface was examined by the double vital staining method. Two µl of preservative-free combination of 1% Rose Bengal and 1% fluorescein dye were instilled into the conjunctival sac, as previously reported [23,24]. The interval between the last complete blink and the appearance of the first corneal black spot in the stained tear film was measured three times and the mean value of the measurements was calculated. Fluorescein and Rose Bengal staining of the ocular surface were also noted and scored. Both fluorescein and Rose Bengal staining scores ranged between 0 and 9 points. Any score above 3 points was regarded as abnormal. For further evaluation of tears, the standard Schirmer test was performed. The standardized strips of filter paper (Showa Yakuhin Kako Co. Ltd., Tokyo, Japan) were placed in the lateral canthus away from the cornea and left in place for 5 min with the eye closed [25]. Readings were recorded in millimeters of wetting for 5 min. A reading of less than 5 mm was referred to as aqueous deficiency.

### Confocal scanning laser microscopy (CSLM)

In vivo confocal scanning laser microscopy was performed on all subjects with a new generation confocal microscope, the Rostock Corneal Software Version 1.2 of the Heidelberg Retina Tomograph II (RCM/HRT II; Heidelberg Engineering GmbH, Heidel berg, Germany). After administration of topical anesthesia with 0.4% oxybuprocaine, the subject’s chin was placed in the chin rest. The objective of the microscope was an immersion lens covered by a polymethylmethacrylate cap (Tomo-Cap; Heidelberg Engineering GmbH). Comfort gel (Bausch&Lomb GmbH, Berlin, Germany) was used as a coupling agent between the applanating lens cap and the conjunctiva. By adjusting the controller, the center of the Tomo-Cap was applanated onto the center of the upper palpebral conjunctiva, and in-vivo digital images of the conjunctiva were visualized directly on the computer screen. When the first superficial cells were seen, the digital micrometer gauge was set at zero, then by pressing on the foot pedal, sequence images were recorded by a charge-coupled device (CCD) color camera (10 frames/s) while gradually moving the focal plane forward into the conjunctival stroma.

The laser source employed in the Heidelberg Retina Tomograph II/ Rostock Corneal Module was a diode laser with a wavelength of 670 nm. Two-dimensional images consisted of 384x384 picture elements, covering an area of 400μm by 400μm. Transversal field of view was captured using the “400 FOV” field lens.

### Conjunctival image analysis

At least three sequences (100 images per sequence) of conjunctival CSLM images were taken for each eye. Three best focused images from the conjunctival epithelium were selected for inflammatory cell counting. After selecting three random non-overlapping frames on each image, the cells were manually marked inside the frame, and the inflammatory cell densities were calculated automatically by the software installed in the machine. The selected images were randomly presented to a masked observer for analysis.

### Conjunctival brush cytology

The brush cytology specimens were obtained after administration of topical anesthesia with 0.4% oxybuprocaine. Central upper palpebral conjunctiva was used for sampling. Conjunctiva was scraped seven times with the Cytobrush-S (Medscand AB, Malmö, Sweden), the examiner holding the brush 2 cm away from the brush end, applying a gentle pressure to the conjunctiva. After sampling, the brushes were immediately placed in 1 ml of Hank’s buffered solution, and the containers were shaken to detach the cells from the brush. The suspended cells were collected using the Millipore filter technique employing filters with 8 μm pore size. The specimens were allocated for the assessment of conjunctival inflammatory cell numbers by DIFF QUIK (Dade Behring, Siemens Healthcare Diagnostice, Deerfield, IL) staining. To be able to delineate the differences in the numbers of inflammatory cells, the epithelial cell counts were ignored. Only the inflammatory cells were counted from 10 non-overlapping fields and the means were calculated for purposes of this study. The inflammatory cell densities were calculated by a single researcher who was masked to the diagnosis and clinical findings. The brush cytology specimens mounted on glass slides also underwent in vivo confocal microscopy. Briefly, after preparation of glass slides, the area of the specimen to undergo confocal microscopy examination (ex vivo) was marked and pressed against the Tomo-cap. The images from photographs of the stained slide and confocal microscopy scans were then compared for cellular morphology.

### Statistical analysis

Data were processed using the Instat, GraphPad software version InStat 3.0 (GraphPad InStat, San Diego, CA). The Kruskal–Wallis test was used to compare the parameters between the AKC subjects and normal controls. The Pearson correlation analysis was used to determine the correlation between the conjunctival inflammatory cell densities assessed by the two techniques as well as the tear quantity, tear stability, and ocular surface vital staining. Pearson correlation analysis was also performed to test the correlation between the corneal inflammatory cell densities, tear stability and ocular surface vital staining. A probability level of less than 5% was considered statistically significant.

## Results

All patients with AKC had active severe AD with pruritus, typical flexural lichenification, papular eruptions, and tendency toward chronically relapsing dermatitis. Personal or family history of atopic disease and “positive” immediate skin reactivity were present in all cases at the time of examination. All patients with AKC had positive skin reactivity to multiple allergens. The most frequent sensitizing allergens in patients with AKC were *Dermatophagoides pteronyssinus*, cedar tree pollen, and *Phleum pratense* pollen. All patients with AKC complained of allergic and dry eye symptomatology, including itchiness, redness, dryness, foreign body sensation, and irritation. There were no age or gender related differences between the patients and the control subjects. At slit lamp observation, all patients had active atopic keratoconjunctivitis as evidenced by conjunctival injection, chemosis, and papillary hypertrophy.

### Conjunctival injection grades

The mean conjunctival injection grade in patients with AKC was 2.5±0.5 points. This value was significantly higher than the mean conjunctival injection grade in the healthy control subjects (0.5±0.5 points) as shown in Table 1 (p<0.05).

**Table 1:** Comparison of tear quantity, tear stability, conjunctival injection grades and ocular surface vital staining scores between patients with atopic keratoconjunctivitis and healthy controls.

**Tear function tests, vital staining scores, and conjunctival injection grades**	**Healthy controls (n=16)**	**AKC patients (n=20)**
Schirmer test (mm)	15.0±2.0	15.0±5.0
BUT (s)	6.6±1.6	3.4±2.4*
Fluorescein score (points)	0.5±0.5	5.5±3.5*
Rose-Bengal (points)	0.1±0.5	4.4±3.6*
Conjunctival injection grade	0.5±0.5	2.5±0.5*

The asterisk indicates a Mann–Whitney p value <0.001.

### Tear function tests and ocular surface vital stainings

The mean tear film break up time was significantly lower in AKC subjects (3.4±2.4 s) compared with the healthy controls (6.6±1.6 s) as shown in Table 1 (p<0.001). The mean fluorescein and Rose Bengal scores in AKC patients were 5.5±3.5 and 4.4±3.6 points, respectively. These values were significantly higher than those measured in the healthy control subjects (0.5±0.5 and 0.1±0.5 points, respectively; p<0.001). There were no statistically significant differences in the mean Schirmer test values between patients with AKC and healthy control subjects (p>0.001).

### Inflammatory cell densities assessed by in-vivo confocal laser scanning microscopy and brush cytology

**Figure 1:**
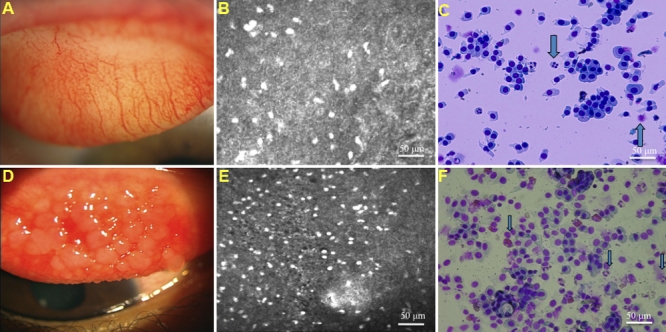
Conjunctival slit-lamp photograph, confocal microscopy images and brush cytology photos from a normal control subject and an AKC patient. **A**: Slit lamp photograph of the upper tarsal conjunctiva in a 26-year-old male healthy control subject. Note the absence of papillary formations. The conjunctival injection grade was 1 point. **B**: Representative confocal scan of the upper tarsal conjunctiva of the same subject. The inflammatory cell density was 256 cells/mm^2^. **C**: Representative photograph of the upper tarsal conjunctival brush cytology specimen of the same subject. The inflammatory cell density was 320 cells/mm^2.^ (Inflammatory cells shown by blue arrows). **D**: Slit lamp photograph of the upper tarsal conjunctiva in a 27-year-old male patient with AKC. Note the cobble stone like papillary formations. The conjunctival injection grade was 3 points. **E**: Representative confocal scan of the upper tarsal conjunctiva of the same patient. The inflammatory cell density was 1,037 cells/mm^2^. **F**: Representative photograph of the upper tarsal conjunctival brush cytology specimen of the same patient. The inflammatory cell density was 856 cells/mm^2^. Inflammatory cells were shown by blue arrows.

**Figure 2:**
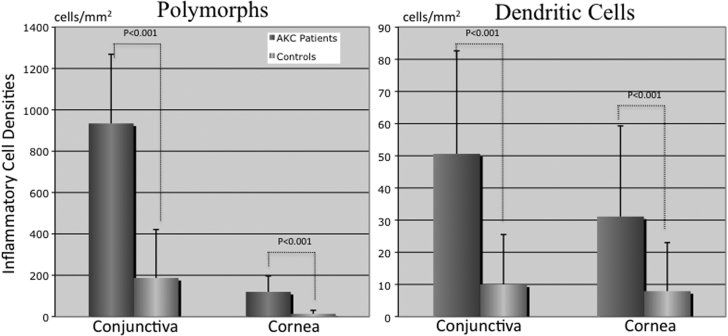
Comparison of conjunctival and cornea inflammatory cell densities between AKC patients and healthy control subjects. Note the higher density of conjunctival and cornea inflammatory cells in AKC patients' eyes when compared to healthy controls eyes (p<0.0001; Mann-Whitney test).

The mean tarsal conjunctival inflammatory cell densities as assessed by the two techniques was significantly higher in patients with AKC compared with healthy control subjects as shown in Table 2 (p<0.001). Figure 1A-C show representative anterior segment photograph, confocal scan, and brush cytology specimen photograph of the tarsal upper palpebral conjunctiva in a 27-year-old male healthy control subject. Figure 1D-F show representative anterior segment photograph, confocal scan, and brush cytology specimen photograph of the tarsal upper palpebral conjunctiva in an age and sex matched patient with AKC. The mean inflammatory cell densities in the confocal scan and brush cytology specimen of the normal subject were 256 and 320 cells/mm^2^, respectively. The mean inflammatory cell densities in the confocal scan and brush cytology specimen of the patient with AKC were 1,037 and 856 cells/mm^2^, respectively. The mean in-vivo confocal microscopy polymorph and dendritic cell densities in the conjunctiva and cornea were significantly higher in patients with AKC compared with healthy control subjects as shown in Figure 2 (p<0.0001).

**Table 2:** Comparison of inflammatory cell densities assessed by in vivo confocal microscopy and conjunctival brush cytology between patients with atopic keratoconjunctivitis and healthy controls.

**Inflammatory cell density (cells/mm^2^)**	**AKC**	**Controls**
Confocal scan	1150±468*	394±158
Brush cytology	837±445*	157±65

Data presented as means±SD. The asterisk indicates a Mann–Whitney p value <0.001.

### The correlation of inflammatory cell densities assessed by in vivo confocal laser scanning microscopy and brush cytology with the tear functions and ocular surface findings

The inflammatory cell densities assessed both by confocal microscopy and brush cytology showed significant linear positive correlations with Schirmer test, fluorescein, and Rose Bengal vital staining scores and conjunctival injection grades. The inflammatory cell densities assessed by both confocal microscopy and brush cytology techniques showed significant linear negative correlations with the tear film break up times as shown in Table 3. The corneal inflammatory cell density assessed by in vivo confocal microscopy showed a significant negative correlation with tear stability and a positive linear correlation with corneal fluorescein staining (Table 4).

**Table 3:** Correlation between tear functions, ocular surface vital staining, conjunctival injection grades, and conjunctival infiltrate density assessed by in vivo confocal microscopy and conjunctival brush cytology.

**Tear function tests, vital staining scores, and conjunctival injection grades**	**Infiltrate Density (CSLM)**	
	**Pearson correlation coefficient**	**p value**
Schirmer test (mm)	0.059	0.778
BUT (s)	−0.471	0.01*
Fluorescein scores (points)	0.522	0.005*
Rose Bengal scores (points)	0.416	0.031*
Conjunctival injection grade	0.622	0.005*
	**Infiltrate Density (Brush cytology)**	
	**Pearson correlation coefficient**	**p value**
Schirmer test (mm)	0.035	0.853
BUT (s)	−0.55	0.01*
Fluorescein scores (points)	0.62	0.005**
Rose Bengal scores (points)	0.522	0.01*
Conjunctival injection grade	0.450	0.01*

The asterisk indicates a p<0.05.

**Table 4:** The correlation between in vivo confocal microscopy corneal inflammatory cell density, tear stability, and ocular surface vital staining.

**Tear function tests and vital staining scores**	**Infiltrate Density (Confocal microscopy)**	
	**Pearson correlation coefficient**	**p value**
BUT (s)	−0.5229	0.0260*
Fluorescein score (points)	0.5788	0.0118*
Rose Bengal score (points)	0.4491	0.0615

### Ex-vivo confocal microscopy scan observations of the brush cytology slide specimens

**Figure 3:**
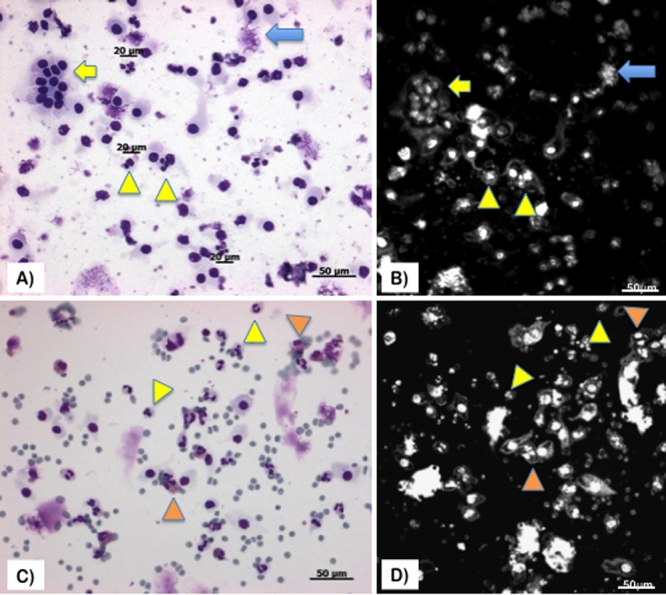
Diff-Quik staining photos and ex vivo confocal microscopy scans of the brush cytology specimens from patients with atopic keratoconjunctivitis. **A** and **B**: Corresponding brush cytology photos and confocal microscopy scans from a representative 12-year-old female patient. Confocal microscopy could effectively discern the nuclear details such as segmentation in polymorphs (yellow arrow heads), epithelial cell clumps (yellow arrows), nuclei in conjunctival epithelial cells (which appeared as round hyperreflective oval bodies) and the mucin blots appeared as hyperreflective bodies with similar/exact shapes resembling the Diff-Quik stained specimens (blue arrows). Corresponding brush cytology photos and confocal microscopy scans from a representative 17 year old male patient are also shown in **C** and **D**. Confocal microscopy could effectively discern the nuclear details such as segmentation in polymorphs (yellow arrow heads), and the double nuclei in eosinophils (orange arrow heads) with similar/exact shapes resembling the Diff-Quik stained specimens.

Corresponding brush cytology photos and confocal microscopy scans from a representative 12-year-old female patient are shown in Figure 3A,B. Confocal microscopy could effectively discern the nuclear details such as segmentation in polymorphs, epithelial cell clumps, and nuclei in conjunctival epithelial cells (which appeared as round hyperreflective oval bodies) and the mucin blots appeared as hyperreflective bodies with similar/exact shapes resembling the Diff-Quik stained specimens. Corresponding brush cytology photos and confocal microscopy scans from a representative 17 year old male patient are also shown in Figure 3C,D. Confocal microscopy could effectively discern the nuclear details such as segmentation in polymorphs, and the double nuclei in eosinophils with similar/exact shapes resembling the Diff-Quik stained specimens.

### Correlation of inflammatory cell densities assessed by in vivo confocal laser scanning microscopy with the inflammatory cell densities assessed by brush cytology

**Figure 4:**
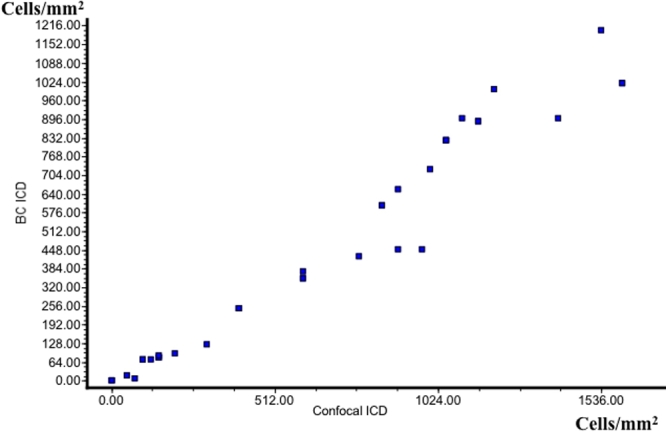
Correlation of conjunctival inflammatory cell densities between in vivo confocal microscopy and brush cytology. Note that a significant positive linear correlation was found between in vivo confocal microscopy and brush cytology inflammatory cell densities. Pearson’s correlation coefficient r=0.97, p<0.0001. Note also that inflammatory cells densities in brush cytology specimens were relatively lower compared to the inflammatory cell densities in the corresponding in vivo confocal microscopy scans which might owe to destruction of some cells during staining and spin down. BC: brush cytology; ICD: inflammatory cell density.

The inflammatory cell densities in the brush cytology samples showed a strong significant positive linear correlation with the inflammatory cell densities calculated from the corresponding conjunctival confocal laser scans (Pearson correlation coefficient 0.97; p<0.0001) as shown in Figure 4.

## Discussion

Atopic keratoconjunctivitis (AKC) is a severe chronic allergic conjunctival disease which is often associated with blinding corneal complications including corneal erosions and ulcers. The conjunctival disease is characterized by papillary formations and infiltration of lymphocytes, neutrophils, mast cells, eosinophils, dendritic cells, and basophils [3,13,26]. Observations from conjunctival biopsy specimens that both neutrophils and eosinophils and their relevant granule proteins are deposited extracellularly in atopic and vernal keratoconjunctivitis suggest a more specific role for both of these cell types in the ocular inflammatory processes of AKC [13]. Neutrophil and eosinophil degranulation resulting in release of neutrophilic elastases and eosinophilic major basic protein, cationic protein, neurotoxins, and peroxidases can induce a grave ocular surface disease in AKC [13,27]. The ocular surface inflammatory status in AKC can be evaluated subjectively and noninvasively by grading the conjunctival injection with slit lamp microscopy or with invasive procedures such as conjunctival biopsy or brush cytology. We previously reported that conjunctival injection grade correlated well with severity of corneal lesions and that percentages of tarsal conjunctival eosinophils and neutrophils increased during corneal ulceration and decreased to almost zero when corneal lesions were not present in a long-term follow up of AKC patients [5,6,21]. Our current study provided further evidence that objective assessment of conjunctival inflammation status by brush cytology correlated well with subjective and objective clinical parameters of ocular surface inflammation and disease such as conjunctival injection grades, vital staining scores, and tear stability. However, although brush cytology is an efficient way to assess conjunctival inflammation, differentiate the type of inflammatory cells with proper staining techniques, and can provide cellular material sufficient to determine mRNA expression of inflammatory proteins or mucins [12,28,29], it is an invasive technique, is irritative, and may predispose to bleeding especially in inflamed conjunctivae.

The confocal scanning laser microscopy is a new, rapid, and non-invasive in vivo clinical examination technique of the eyelids, meibomian glands, cornea, and conjunctiva [14-21,30]. Of importance in the current study were our first time observations that inflammatory cell density assessed by confocal microscopy correlated strongly and significantly with inflammatory cell densities evaluated by conjunctival brush cytology. The corneal inflammatory cell density assessed by in vivo confocal microscopy also showed a significant negative correlation with tear stability and a positive linear correlation with corneal fluorescein staining suggesting that in vivo confocal microscopy could effectively reflect the severity of corneal disorder in AKC.

These observations suggest that confocal microscopy may very well be employed as an objective quantitative adjunctive ocular surface examination procedure beside slit lamp examination, especially for the delineation of ocular surface inflammation status. Its application to the diagnosis, follow up, and evaluation of the ocular surface disease in other entities associated with ocular inflammation such as Sjogren’s syndrome, Stevens Johnson syndrome, graft versus host disease, pemphigoid, rosacea, and infectious disorders may also be rewarding. In this study, we also employed an ex vivo application of confocal microscopy on brush cytology specimens and compared the morphology of the corresponding areas in brush cytology photographs and confocal scans. Confocal microscopy could effectively discern the nuclear details such as segmentation in polymorphs, double nuclei in eosinophils, nuclei in conjunctival epithelial cells (which appeared as round hyperreflective oval bodies), and the mucin blots appeared as hyperreflective bodies with similar/exact shapes resembling the Diff-Quik stained specimens. We believe that it was possible to discern these details since brush cytology specimens are mounted on a transparent glass slide and usually consist of a single layer of cells collected on the glass slide after centrifugation. The in vivo confocal scans of inflammatory cells in the conjunctiva lacked nuclear detail but could be discerned mainly by size. Although with its current state, the in vivo applications of this new technology can differentiate between only dendritic cells and polymorphs based on morphology and size, it is our belief that further improvements in this technology will provide higher resolution ultrastructural in vivo images enabling us to differentiate between major inflammatory cell types in ocular allergy including neutrophils, eosinophils, and mononuclear cell types in the near future. It should be noted that confocal microscopy showed more cell counting values compared with brush cytology in this study. We believe that confocal microscopy provides a more realistic value in relation to inflammatory cell number in vivo. On the other hand, while brush cytology enables differentiation between inflammatory cells through staining of the slides, some amount of epithelial and inflammatory cells are destroyed during the brush and spin down procedures. This may explain the differences in inflammatory cell numbers with both techniques. The findings of this study may be limited because all subjects were using topical antiallergic and steroid eye drops owing to the severity of the disease process; a washout period was not allowed by the ethics board. Studying the naïve ocular surface in ocular allergy is a challenging issue because most patients also have undergone treatments before referral to specialty centers. We tried to tackle these confounding issues by recruiting only those patients who used the same nonpreserved eye drops with the same duration and frequency of instillations. Despite our inability to study the naïve conjunctival status in AKC which remains to be investigated in future trials, the presence of striking inflammatory cell differences between patients with AKC and healthy control subjects is still noteworthy and important. The current study provides interesting new data regarding the relation of inflammatory cell findings between in vivo confocal microscopy and brush cytology.

In summary, inflammatory cell density seems to be an important parameter of confocal laser scanning microscopy which correlated well with subjective clinical signs of inflammation and inflammatory cell numbers in brush cytology. Confocal microscopy can be instituted instead of brush cytology for the study and follow up of the severity of ocular surface disease in clinical study protocols where quantitative assessment of inflammatory status is essential.
